# Frontiers in zeolites: towards establishing a new discipline of condensed matter chemistry—an interview with Ruren Xu

**DOI:** 10.1093/nsr/nwac056

**Published:** 2022-03-25

**Authors:** Wenfu Yan

**Affiliations:** Professor, Jilin University

## Abstract

In recent decades, the application of zeolite has been extended to many sustainable processes. Professor Ruren Xu (徐如人) of Jilin University is a leader within Chinese, Asian and worldwide zeolite communities, as well as the founder of the inorganic synthesis discipline in China and the first person in the world to propose the scientific discipline of modern inorganic synthetic chemistry. Professor Xu started his scholarly research on zeolites in the mid-1970s. He focused initially on crystallization and mechanisms of zeolite formation. In the 1980s, he gradually shifted his research to the exploration of microporous materials with novel frameworks and compositions. In 1984, he outlined new directions in the synthesis of zeolites and placed emphasis on the ‘heteroatom concept’, which turned out to be very influential and fruitful for the subsequent development of heteroatom-containing zeolite catalysts. In the following years, he and his group systematically developed new solvothermal routes for zeolite synthesis. In the late 1990s, Xu started to think about the rational synthesis of zeolites, a major challenge for zeolite as well as inorganic synthesis in general. His group developed several effective strategies for the rational design and synthesis of zeolitic materials. He is the chairman of the 15th International Zeolite Conference (15th IZC) held in 2007 for the first time in China. Because of his significant contribution to zeolite science in China, he received the National Zeolite Lifetime Achievement Award of China in 2017.

*NSR* recently interviewed Professor Xu about the current status and future prospects of zeolites and related porous materials. This interview is dedicated to Professor Xu on his 90th birthday, in recognition of his seminal contribution to zeolite science, modern inorganic synthetic chemistry and the new discipline of condensed matter chemistry, which was first suggested by Professor Xu in 2018.

***NSR:*** Could you please briefly introduce the history of the science of zeolites and related porous materials and the contribution by the Jilin group to the development of zeolite science?

***Xu***: The term ‘zeolite’ was first coined by Swedish Mineralogist Axel Fredrik Cronstedt in 1756 to name a new type of mineral that produced a large amount of steam from water when heated up rapidly. In the 1950s, R.M. Milton from the Union Carbide Corporation made the first synthetic zeolites under hydrothermal conditions. Since then, great efforts have been made to discover new types of zeolites and related porous materials. Besides zeolites with pore sizes smaller than 2.0 nm, mesoporous materials with pore sizes between 2.0 and 50 nm, including mesoporous polymer and mesoporous carbon, porous metal-organic-frameworks (MOFs), and porous organic materials such as porous aromatic frameworks (PAFs), have greatly extended the compositions of porous materials, making porous materials an important area in materials science.

The group at the State Key Laboratory of Inorganic Synthesis and Preparative Chemistry at Jilin University has been working on zeolites since the mid-1970s, initially focusing on the crystallization and formation mechanism of zeolites followed by the synthesis of heteroatom-doped zeolites and open frameworks with new tetrahedral elements and building units. The most publicized examples include JDF-20, a microporous aluminophosphate with the largest 20-membered ring, AlPO-CJB1, the first aluminophosphate sieve with Brönsted acidity, and MAPO-CJ40, a heteroatom-stabilized chiral framework of aluminophosphate molecular sieve with the zeolite structure of JRY, the first zeotype structure discovered by scientists from China.

**Figure 1:**
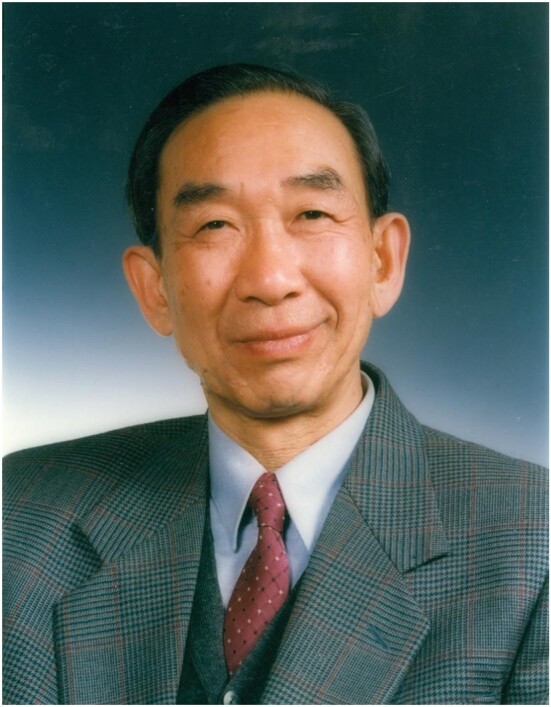
Professor Ruren Xu at Jilin University; a leader in the zeolite community and the founder of the inorganic synthesis discipline in China *(courtesy of Professor Ruren Xu)*.

International colleagues in the field of zeolite research widely consider our group to be leaders in the discovery of numerous compounds with abundant structure types and compositions, referred to as the third milestone in the field. Our group is therefore widely referred to as ‘the Jilin Group’ by international colleagues, and is considered to be an important team in the international zeolite research community. Two of the members of this group are Professor Jihong Yu (于吉红) of Jilin University and Professor Fengshou Xiao (肖丰收) now at Zhejiang University. Professor Jihong Yu has had great success in establishing the methodologies for the rational design and synthesis of zeolites and revealing the crystallization mechanism of zeolites, especially the discovery of the hydroxyl free radicals involved in the crystallization mechanism of zeolites. She has also developed new applications of zeolites to catalysis, separation and energy storage. Professor Fengshou Xiao has successfully developed a green synthesis route for zeolites, i.e. a template- and solvent-free route, and highly efficient catalysis systems with synergistic functionalities. In the 1990s, two scholars, Dr. Qisheng Huo (霍启升) and then Dr. Dongyuan Zhao (赵东元) from our group, joined the group of Professor G.D. Stucky at UC Santa Barbara. They became pioneers in the field of ‘mesostructured materials’. Professor Shilun Qiu (裘式纶) made contributions to the early development of MOFs, the membrane of covalent organic frameworks (COFs); and PAFs and COFs with exceptional adsorption, separation and catalysis. Professor Dongyuan Zhao (赵东元) and his group developed, for the first time in the early years of the new century, a new self-assembly route of organic-organic components for the construction of ordered polymer and carbon-based materials, which has been applied to macro-molecules catalysis, adsorption-separation, nanoscale assembly and biochemical systems. Their achievements have significantly advanced the field of porous materials and made great contributions to the field of zeolites.

***NSR***: In recent years, you have been working tirelessly to develop the fields of condensed matter chemistry and condensed matter engineering. Could you please give a brief overview of condensed matter chemistry and engineering, and their relationship to other modern chemistry sciences?

***Xu***: Since the early 1800s, more than 193 million organic and inorganic substances, including alloys, coordination compounds, minerals, mixtures, polymers and salts, have been discovered and presented in the scientific literature. These substances are either natural or human-made via chemical reactions, whereas chemical reactions are the core of the science of chemistry. The traditional thinking has been that the main components in all chemical reactions are molecules, atoms and/or ions, and virtually no attention has been paid to the states of the reactants, which are generally in condensed states like solids, liquids and mesostructures, or even in complex living organisms. Therefore, the processes and products of chemical reactions should not be determined solely by the structure and composition of these basic species but also by the complex, and possibly multilevel-structured, physical and chemical environment, together referred to as the *condensed state* [1–3]. That is, the relevant matters in the condensed state should be the main bodies of chemical reactions; this is applicable not only to solids and liquids but also to gas molecules, as reactions among gas molecules can take place only in the presence of catalysts in specific condensed states, or after their state transition under extreme reaction conditions. The reaction process, the mechanism and the reaction products are dictated, possibly predominantly by the composition and the multilevel structure of the catalysts in condensed matter states.

To achieve a more realistic view of chemical reactions, we need to establish a new chemistry discipline, i.e. condensed matter chemistry, to gain an improved understanding of the actual reaction processes of chemical reactions in the condensed state and to establish associations among functionalities, multilevel structures and properties of the reactants in complex environments. I anticipate that big data and artificial-intelligence-based machine-learning techniques may play indispensable roles as we work to derive general principles and rules from the available data of reactants, reactions and products, along with reaction conditions, possibly guided by principles and knowledge from the science of condensed matter physics.

The processes and products of chemical reactions should not be determined solely by the structure and composition of these basic species but also by the complex, and possibly multilevel-structured, physical and chemical environment, together referred to as the condensed state.

—Ruren Xu

***NSR:*** In your opinion, what are the important frontiers in the field of zeolites and related porous materials?

***Xu:*** I think the important frontiers in the field of zeolites and related porous materials include: (i) theoretical study of the synthesis, characterization and functionality of zeolites and related porous materials; (ii) development of new zeolites and porous materials with desired functionalities; and (iii) development of the science of condensed matter engineering for porous materials, which involves structure design and rational construction at the condensed state level (i.e. rational synthesis, preparation and self-assembly).

***NSR:*** Could you please briefly describe the relationship between zeolites and related porous materials, as well as condensed matter chemistry?

***Xu***: Taking zeolites with specific catalytic performance as an example, the synthesis and preparation stages, as well as the catalysis process, all involve complex condensed matter chemistry. The composition and multilevel structure in the condensed state of the catalyst, the local environment, and the interactions among the reactants enabled by the catalytic sites, determine the catalysis mechanism, process, yield, side-reaction and types of products. During the crystallization process of zeolites under hydro/solvothermal conditions, the composition and structure of the liquid phase will affect the condensation reaction between species, the gelation process, the gel composition, gel crystallization in the presence of the template and the crystallinity of the products, among a few other things. These issues need to be studied at the level of condensed matter chemistry. Studying these issues will push forward the development of condensed matter chemistry.

***NSR:*** How do you achieve the rational design and synthesis of zeolites and related porous materials with specific functionalities?

***Xu***: One possible way is to establish the relationship among functionalities in the condensed state, multilevel structure and construction of matter through the development of condensed matter engineering, coupled with mining and modeling big data using artificial intelligence techniques. With such relationships established, we can design the composition and structure in the condensed state of zeolites with specific functionalities and accomplish the rational synthesis and precise preparation/modification of the relevant zeolites. Regarding the development of condensed matter engineering, it was started in the early 1990s via the project ‘Construction of Molecular Engineering’ sponsored by the National Pandeng (攀登, means climb) Project. The principal investigators included our group, Professor Youqi Tang's (唐有祺) group of Peking University and four other universities and two research institutes. The projects went on for 25 years with the first 10 years supported by the National Pandeng Project and the next 15 years by the ‘973 Project’. Through these projects we have gained considerable knowledge and experience, and built a foundation for the current development of condensed matter engineering.

***NSR:*** What suggestions do you have for young researchers working in the field of zeolites and related porous materials?

***Xu***: Zeolites and related porous materials are extremely important materials with great potential for application. Here, I encourage young researchers working in this field to consider the issues from the perspective of condensed matter physical science when they develop new types of porous materials with new functionalities, explore new application areas of porous materials, and investigate the rational construction and precise preparation of matter with specific multilevel structures in condensed states. This new knowledge will serve as the basis and direction for the rapid development of condensed matter chemistry in other fields of chemistry.
